# Global Gene Expression Associated with Hepatocarcinogenesis in Adult Male Mice Induced by *in Utero* Arsenic Exposure

**DOI:** 10.1289/ehp.8534

**Published:** 2005-10-06

**Authors:** Jie Liu, Yaxiong Xie, Danica M.K. Ducharme, Jun Shen, Bhalchandra A. Diwan, B. Alex Merrick, Sherry F. Grissom, Charles J. Tucker, Richard S. Paules, Raymond Tennant, Michael P. Waalkes

**Affiliations:** 1 Inorganic Carcinogenesis Section, Laboratory of Comparative Carcinogenesis, National Cancer Institute at the National Institute of Environmental Health Sciences, and; 2 National Center For Toxicogenomics, National Institute of Environmental Health Sciences, National Institutes of Health, Department of Health and Human Services, Research Triangle Park, North Carolina, USA; 3 Basic Research Program, Science Applications International Corporation, National Cancer Institute, National Institutes of Health, Department of Health and Human Services, Frederick, Maryland, USA

**Keywords:** Agilent mouse oligo 22K microarray, arsenic, hepatocellular carcinoma, real-time RT-PCR, transplacental exposure, Western blotting

## Abstract

Our previous work has shown that exposure to inorganic arsenic *in utero* produces hepatocellular carcinoma (HCC) in adult male mice. To explore further the molecular mechanisms of transplacental arsenic hepatocarcinogenesis, we conducted a second arsenic transplacental carcinogenesis study and used a genomewide microarray to profile arsenic-induced aberrant gene expression more extensively. Briefly, pregnant C3H mice were given drinking water containing 85 ppm arsenic as sodium arsenite or unaltered water from days 8 to 18 of gestation. The incidence of HCC in adult male offspring was increased 4-fold and tumor multiplicity 3-fold after transplacental arsenic exposure. Samples of normal liver and liver tumors were taken at autopsy for genomic analysis. Arsenic exposure *in utero* resulted in significant alterations (*p* < 0.001) in the expression of 2,010 genes in arsenic-exposed liver samples and in the expression of 2,540 genes in arsenic-induced HCC. Ingenuity Pathway Analysis revealed that significant alterations in gene expression occurred in a number of biological networks, and Myc plays a critical role in one of the primary networks. Real-time reverse transcriptase–polymerase chain reaction and Western blot analysis of selected genes/proteins showed > 90% concordance. Arsenic-altered gene expression included activation of oncogenes and HCC biomarkers, and increased expression of cell proliferation–related genes, stress proteins, and insulin-like growth factors and genes involved in cell–cell communications. Liver feminization was evidenced by increased expression of estrogen-linked genes and altered expression of genes that encode gender-related metabolic enzymes. These novel findings are in agreement with the biology and histology of arsenic-induced HCC, thereby indicating that multiple genetic events are associated with transplacental arsenic hepatocarcinogenesis.

Inorganic arsenic is an environmental pollutant. Arsenic exposure in humans comes mainly from consumption of drinking water contaminated with inorganic arsenic [National Research Council (NRC) 1999], but elevated exposure can occur from burning of coal containing high levels of inorganic arsenic (Liu et al. 2002). Epidemiologic studies have demonstrated that chronic arsenic exposure causes tumors of the skin, urinary bladder, lung, liver, prostate, kidney, and possibly other sites [International Agency for Research on Cancer (IARC) 1987; Morales et al. 2000; NRC 1999]. Until recently, inorganic arsenic was considered a “paradoxical” human carcinogen because of strong human evidence but limited evidence for animal carcinogenesis (IARC 1987; Waalkes et al. 2004a, 2004b). Indeed, negative results had been obtained for inorganic arsenic carcinogenesis in mice, rats, hamsters, rabbits, dogs, and monkeys (IARC 1987; Kitchin 2001). Recently, important advances have been made in the development of rodent models of inorganic arsenic carcinogenesis. These include skin cancer models in which inorganic arsenic acts as a co-promoter with 12-*O*-tetradecanoyl phorbol-13-acetate in Tg.AC mice (Germolec et al. 1998), or as a co-carcinogen with ultraviolet irradiation in hairless mice (Rossman et al. 2001). In addition, our group has developed a transplacental model in which short-term exposure to inorganic arsenic *in utero* produces a variety of internal tumors in the offspring when mice reach adulthood (Waalkes et al. 2003, 2004b). These tumors include cancers of the liver, lung, ovary, and adrenal induced in adulthood by transplacental exposure to arsenic (Waalkes et al. 2003, 2004b).

The brief period of exposure (10 days) to inorganic arsenic, which was sufficient for it to be a complete transplacental carcinogen in mice, is not only alarming but points to a clear period of high sensitivity to the metalloid. Indeed, gestation is a period of high sensitivity to chemical carcinogenesis in general in both rodents and probably humans (Anderson et al. 2000). Inorganic arsenic can readily cross the placenta and enter the fetal system (Chattopadhyay et al. 2002; Concha et al. 1998; NRC 1999). Thus, in human populations exposed to inorganic arsenic, it is reasonable to assume that exposure during gestation does occur, and that the carcinogenesis sensitivity observed in rodents may predict similar effects in humans.

Hepatocellular carcinoma (HCC) has been identified as a tumor type associated with arsenic exposure in humans (Centeno et al. 2002; Chen and Ahsan 2004; Chen et al. 1997; Zhou et al. 2002). HCC and non-alcoholic liver cirrhosis and ascites are leading causes of mortality in the arsenic endemic area of Guizhou, China (Liu et al. 2002; Zhou et al. 2002). Also, hepatomegaly and liver diseases are commonly associated with arsenic exposure through the drinking water in West Bengal, India (Santra et al. 2000). In accord with human data, transplacental exposure to inorganic arsenic in mice induced a marked, dose-related increase in HCC formation in male offspring in both an initial (Waalkes et al. 2003) and a subsequent followup study (Waalkes et al. 2004a, 2004b). *In utero* arsenic exposure also caused a variety of hepatic genes to be aberrantly expressed, including genes probably critical to the carcinogenic process, as evidenced by analysis of liver samples taken at autopsy during the initial transplacental study with sodium arsenite (Liu et al. 2004; Waalkes et al. 2004a). This initial genomic analysis was limited to individual genes of interest (Waalkes et al. 2004a) or to a limited number of genes (588) in a custom array (Liu et al. 2004).

Our second transplacental arsenic carcinogenesis study in mice (Waalkes et al. 2004b) confirmed the hepatocarcinogenic potential of gestational arsenic exposure seen in the initial study (Waalkes et al. 2003). It also provided a unique opportunity to perform a genomewide analysis through the National Center for Toxicogenomics using the Agilent 22K chip arrays. Changes in expression of specific genes of interest were confirmed by real-time reverse transcriptase–polymerase chain reaction (RT-PCR) and Western blot analysis. This study confirmed and extended our initial observations, but it also revealed a number of novel pathways and gene expression alterations potentially associated with hepatocarcinogenesis induced by transplacental arsenic exposure.

## Materials and Methods

### Chemicals.

Sodium arsenite (NaAsO_2_) was obtained from Sigma Chemical Co. (St. Louis, MO) and dissolved in the drinking water at 85 mg arsenic/L (85 ppm). The Agilent 22K mouse oligo array was obtained from Agilent Technologies (Palo Alto, CA). Monoclonal antibodies against α-fetoprotein (sc-8399), K-ras (sc-30), c-myc (sc-42), estrogen receptor (ER)- α (sc-8002), β-actin (sc-1616) and poly-clonal antibodies against cyclin D1 (sc-753), cdk4 (sc-601), and endothelial growth factor receptor (EGFR, sc-03) were obtained from Santa Cruz Biotechnology (Santa Cruz, CA). Monoclonal antibodies against proliferating cell nuclear antigen (PCNA, 610664), cytokeratin 5/8 (550505), and plasminogen activator inhibitor-1 (PAI-1, 612024) were purchased from BD Biosciences (San Jose, CA). All other chemicals were commercially available and of reagent grade.

### Animal treatment and sample collection.

We conducted the present study using liver tumors and nontumorous normal liver samples collected in the second of a series of transplacental arsenic carcinogenesis studies in mice for which pathological results have already been reported (Waalkes et al. 2004b). In this study timed pregnant C3H mice were given drinking water *ad libitum* containing 85 ppm arsenic as sodium arsenite or unaltered water from days 8–18 of gestation. Offspring were weaned at 4 weeks, then randomly placed into separate groups (*n* = 25 from 10 dams/group) according to maternal exposure level and observed for up to 104 weeks. Animal care was provided in accordance with the *Guide on the Care and Use of Animals* (Institute of Laboratory Animal Resources 1996), and the Institutional Animal Care and Use Committee approved the study proposal. Animals used in this study were treated humanely and with regard for the alleviation of suffering. Samples of large tumors, later confirmed as HCC, and normal surrounding liver tissues were taken at autopsy, snap frozen in liquid nitrogen, and compared with normal liver samples from contemporaneous controls. A total of 17 samples from male mice were analyzed, including 5 inorganic arsenic–induced HCC samples (designated As-HCC), 5 samples of surrounding nontumorous, normal liver tissues from arsenic-treated mice (As-Normal), and 7 samples of age-matched control mouse liver (Control).

### Microarray analysis.

We isolated total RNA from liver samples with TRIzol agent (Invitrogen, Carlsbad, CA), followed by purification and DNase-I digestion with RNeasy columns (Qiagen, Valencia, CA). We evaluated the quality of the RNA using an Agilent 2100 Bioanalyzer (Agilent Technologies). Total RNA was amplified using the Agilent Low RNA Input Fluorescent Linear Amplification Kit protocol. Starting with 500 ng of total RNA, cyanine (Cy)3- or Cy5-labeled cRNA was produced according to the manufacturer’s protocol. For each two-color comparison, 750 ng each of Cy3- and Cy5-labeled cRNA was mixed and fragmented using the Agilent In Situ Hybridization Kit protocol (Agilent Technologies). We performed hybridizations on Agilent mouse 22K oligo assay for 17 hr in a rotating hybridization oven using the Agilent 60-mer oligo microarray processing protocol. Slides were washed as indicated in this protocol and then scanned with an Agilent Scanner. Data were obtained using the Agilent Feature Extraction software (version 7.5), employing defaults for all parameters. Two hybridizations with fluor reversals were performed for each RNA sample from each group.

### Rosetta Resolver.

Images and GEML (gene expression markup language) files, including error and *p*-values, were exported from the Agilent Feature Extraction software and deposited into the Rosetta Resolver system (version 4.0, build 4.0.1.0.7.RSPLIT) (Rosetta Biosoftware, Kirkland, WA). We combined the resultant ratio profiles. Intensity plots were generated for each ratio experiment, and genes were considered “signature genes” if the *p*-value was < 0.001.

### Ingenuity Pathways Analysis.

We further analyzed the signature genes with Ingenuity Pathways Analysis (Ingenuity Systems, Redwood City, CA; http://www.ingenuity.com), a web-delivered application that enables biologists to discover, visualize, and explore relevant networks significant to their experimental results, such as gene expression array data sets. A data set containing gene identifiers and their corresponding expression values such as fold-changes and *p*-values was uploaded as a tab-delimited text file. Each gene identifier was mapped to its corresponding gene object in the Ingenuity Pathways Knowledge Base. A fold change cutoff of 1.5-fold and *p* < 0.001 was set to identify genes whose expression was differentially regulated. These genes, called “focus genes,” were then used as the starting point for generating biological networks. To start building networks, the application queries the Ingenuity Pathways Knowledge Base for interactions between focus genes and all other gene objects stored in the knowledge base, then generates a set of networks with a network size of approximately 35 genes or proteins. Ingenuity Pathways Analysis then computes a score for each network according to the fit of the user’s set of significant genes. The score is derived from a *p*-value and indicates the likelihood of the focus genes in a network being found together because of random chance. A score of 2 indicates a 1 in 100 change that the focus genes are together in a network because of random chance. Therefore, scores of 2 or higher have at least a 99% confidence level of not being generated by random chance alone. Biological functions are then calculated and assigned to each network.

### Real-time RT-PCR analysis.

We used real-time RT-PCR analysis to quantify the levels of expression of the selected genes (Liu et al. 2004). The forward and reverse primers for selected genes were designed using ABI Primer Express software (Applied Biosystems, Foster City, CA). Total RNA was reverse transcribed with MuLV reverse transcriptase and oligo-dT primers, then subjected to real-time PCR analysis using SYBR green PCR master mix (Applied Biosystems, Cheshire, UK). The relative differences in expression between groups were determined using cycle time (C_t_) values as follows: The C_t_ values of the genes of interest were first normalized with β-actin of the same sample; then the relative differences between control and treatment groups were calculated and expressed as relative increases, setting the control as 100%. Assuming the C_t_ value reflects the initial starting copy and there is 100% efficiency, a difference of one cycle is equivalent to a 2-fold difference in starting copy.

### Western blot analysis.

Tissues were homogenized (1:20, w:v) in PER-Tissue Protein Extraction buffer (Pierce, Rockford, IL) containing freshly added protease inhibitor cocktail (Calbiochem, La Jolla, CA) and 100 μM phenylmethylsulfonyl fluoride. Cytosols were prepared by centrifugation at 15,000 × *g* for 10 min at 4° C. Protein concentrations were determined using the dye-binding assay (Bio-Rad, Hercules, CA). Total protein (30 μg) was subjected to electrophoresis on NuPAGE Bis-Tris gels (4–12%) (Invitrogen, San Diego, CA), followed by electrophoretic transfer to nitrocellulose membranes at 30 V for 1 hr. Membranes were blocked in 5% dried milk in TBST (15 mM Tris–HCl, pH 7.4, 150 mM NaCl, and 0.05% Tween 20) for 2 hr, followed by incubation with the primary antibody (1:200 to 1:1,000) in Blotto (Pierce, Rockford, IL) overnight at 4° C. After washes with TBST, the membranes were incubated in horse radish peroxidase–conjugated secondary antibody (1:4,000 to 1:10,000) for 1 hr and washed with TBST 3 times. Immunoblots were visualized using SuperSignal chemiluminescent substrate (Pierce, Rockford, IL).

### Statistics.

For microarray analysis, we cross-hybridized samples with Cy3 and Cy5. For Ingenuity Pathways Analysis, we used a Fisher’s exact test to calculate a *p*-value to determine the probability that the biological function assigned to that network could be explained by chance alone. For real-time RT-PCR analysis, means and SEM of individual samples (*n* = 5–7) were calculated. For the comparisons of gene expression between two groups, Student’s *t*-test was performed. For comparisons among three or more groups, data were analyzed using analysis of variance, followed by Duncan’s multiple range test. The level of significance was set at *p* < 0.05 in all cases.

## Results

### Microarray analysis of aberrantly expressed genes.

**Figure 1:**
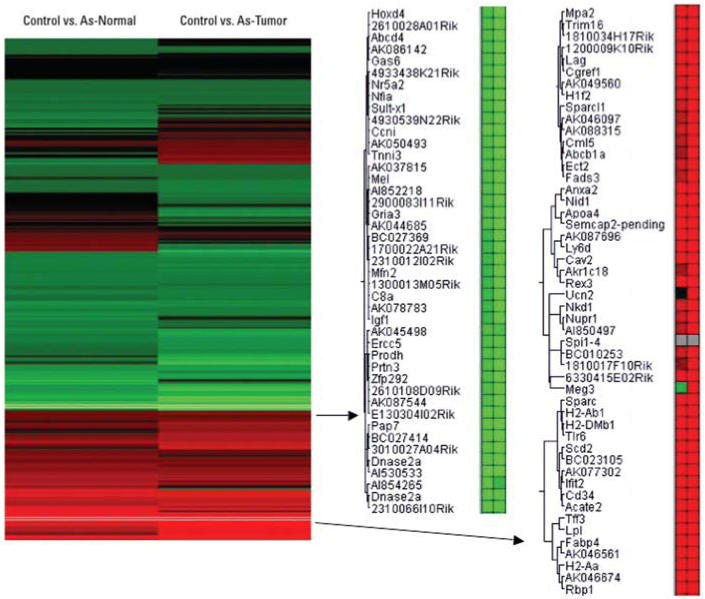
Altered gene expression in adult male mice exposed to arsenic *in utero*. The significantly altered genes under criteria of > 1.5-fold difference and *p* < 0.001 were clustered for comparison. Arsenic-exposed normal liver samples and arsenic-induced liver tumors are compared to control livers. Increased gene expression is shown in red, and decreased gene expression is shown in green. Gene symbols and accession numbers are from GenBank (http://www.ncbi.nih.gov/GenBank/).

The hepatocarcinogenic effects of inorganic arsenic have been verified in a study in which HCC developed in high incidence in male mice after *in utero* exposure (Waalkes et al. 2004b). Thus, total RNA was isolated from samples of control mouse liver, arsenic-exposed nontumorous normal liver, and arsenic-induced HCC taken at autopsy, and subjected to microarray analysis. Under the criteria of *p* < 0.001 by the Rosetta Resolver (version 4.0) system, the expression of 2,010 genes was significantly altered in arsenic-exposed normal liver samples compared with control, and 2,540 genes were altered in arsenic-induced HCC. The clustering analysis of these gene alterations clearly showed they generally occur in the same direction (increase or decrease) but are much more pronounced in arsenic-induced tumors than normal surrounding tissue from arsenic exposed mice (Table 1, Figure 1). Examples of a cluster showing increased genes (shown in red) and an example of a gene cluster showing decreased genes (green) are shown in Figure 1. Generally, gene alterations seen in the present study are largely consistent with the previous, more limited gene analysis (Liu et al. 2004) of our first transplacental arsenic carcinogenesis study (Waalkes et al. 2003). The significant gene expression alterations (*p* < 0.001 and 1.5-fold cutoff) were further selected for Ingenuity Pathway Analysis.

### Ingenuity Pathways Analysis.

**Figure 2:**
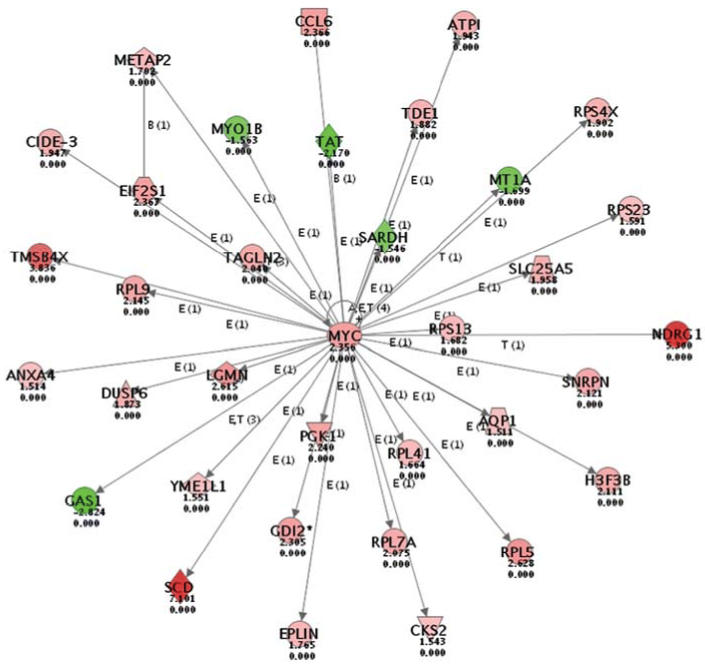
The Ingenuity Pathways Analysis of gene expression changes (detailed in “Material and Methods”). The network number 1 from arsenic-induced HCC and control liver samples is depicted. The central role of MYC activation in transplacental arsenic carcinogenesis is illustrated. Red indicates increases in gene expression, and green indicates decreases.

To further analyze the biological significance of these alterations in gene expression, we used the Ingenuity Pathways Analysis; an example of the highest score in the analysis from arsenic-induced tumors (Figure 2) is depicted. The intensity of the node color indicates the degree of increased (red) or decreased (green) expression in arsenic-induced HCC. Biological functions were assigned to each gene network by using the findings extracted from the published scientific literature and stored in the Ingenuity Pathways Knowledge Base (http://www.ingenuity.com/products/PathwaysKnowledge.pdf) and are ranked according to the significance of that biological function to the network. The activation of *c-myc* oncogene and its central role in related pathway alterations are consistent with the published literature on arsenic (Chen et al. 2001b), and could be a major component of its hepatocarcinogenic potential.

### Real-time RT-PCR analysis of aberrantly expressed genes.

To confirm and extend microarray results, we performed real-time RT–PCR analysis of selected genes using individual samples from control mouse liver, arsenic-exposed nontumorous livers, and arsenic-induced HCC. Generally, real-time RT-PCR confirmed microarray results with > 90% concordance but tended to show more pronounced changes than microarray analysis (Table 1). For HCC biomarkers, alpha-fetoprotein (AFP, 19-fold), plasminogen activator inhibitor-1 (PAI-1, 9-fold), cytokeratin 8 (6-fold), cytokeratin 18 (3-fold) and cytokeratin 19 (11-fold), were dramatically increased in arsenic-induced HCC. The oncogenes *c-myc, c-met, k-ras* and the neoplastic progression protein-3 (Npn3) were also increased 2- to 3-fold in arsenic-induced HCC and nontumorous, normal liver from arsenic exposed mice, while the tumor suppressors BRCA1 and BRCA2 decreased 30–50%.

The positive cell cycle regulators cyclin D1 (5-fold), cyclin E (5-fold), cdk2na (14-fold), cdk2nb (6-fold), cdk4 (4-fold), and PCNA (3-fold) were increased as a result of arsenic exposure, while the cell cycle negative regulator p16 decreased 40%. The expression of insulin-like growth factor-1 (IGF-1, 60% of control) was decreased, while the expression of IGF-2 was increased 4-fold. The IGF-binding proteins (IGFBP1, 9-fold), IGFBP3 (3-fold), and IGFBP5 (2.4-fold) were all increased to some extent.

Arsenic produces oxidative stress within cells as a possible mechanism of its toxicity (Liu et al. 2001a). Stress-related genes, including various glutathione *S*-transferases (GST-alpha, GST-mu, GST-pi, and GST-theta) and early growth response protein 1 (EGR1), increased 2.5- to 4.5-fold. In addition, Cu,Zn-superoxide dismutase (SOD1) and ceruloplasmin (Cu-binding protein) were also increased approximately 3-fold by arsenic. In comparison, no major changes occurred in the expression of heme oxygenase-1 (HO-1), a biomarker for arsenic-induced oxidative stress (NRC 1999), and the expression of metallothionein-1 (MT-1) actually decreased 50%.

A feminized pattern of metabolic enzymes has been noted in male livers of mice bearing arsenic-induced HCC after *in utero* exposure (Waalkes et al. 2004a). Marked elevation in expression of the female predominant cytochrome P450 (CYP)2A4 (25-fold), and significant increases (2- to 3-fold) of female dominant CYP2B9 (2.6-fold) and CYP2D9 were evident. In contrast, expression of the male dominant CYP7B1, CYP2F2, and CYP41 were all decreased by approximately 50% by arsenic exposure. Further evidence for feminization includes increased expression of aldoketo reductase 1c18 (Akr1c18) and hydroxysteroid 17β dehydrogenase 7 (HSD 17β7), as well as ER-α linked overexpression of trefoil factor 3 (TFF3) and cysteine-rich protein 61 (Cyr61). No apparent changes occurred for CYP2C39, CYP2E1, CYP2J5, CYP3A25, and CYP4A14 (data not shown). Interestingly, the enzymes for lipid metabolism such as lipoprotein lipase (Lpl, 33-fold), cytosolic acyl-CoA thioesterase (Cte1, 5-fold), and proteasome 26S subunit ATPase-3 (Pmsc3, 4-fold) were increased. Conversely, betaine homocysteine methyl-transferase (BHMT, 60% of control), serum amyloid 3 (Saa3, 30% of control), and sulfotransferase-related protein 2 (SULT-X2, 1% of control) were decreased.

For cell–cell interaction genes and genes involved in signal transduction, marked increases occurred in annexin A2 (48-fold), nidogen 1 (50-fold), E-cadherin (11-fold), and β-catenin (7-fold) in arsenic-induced HCC. Moderate increases in the ras homolog gene family U (Rhou, 4-fold), in fibronection (CTGF, 4-fold), and in prostaglandin-endoperoxide synthase 2 (Ptgs-2, 5.5-fold) were also evident. In contrast, the prolactin receptor (Prlr, 40% of control) and the epidermal growth factor receptor (Egfr, 40% of control) were decreased as a result of *in utero* exposure to inorganic arsenic.

### Western blot analysis of aberrantly expressed proteins.

**Figure 3:**
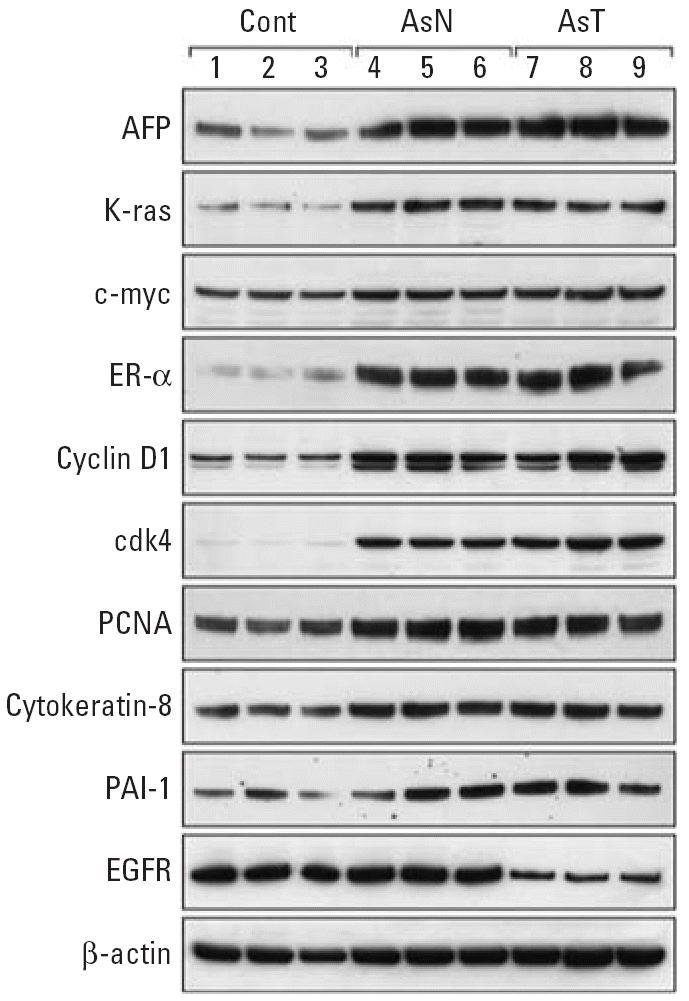
Western blot analysis of selected proteins in control mouse livers (Cont), arsenic-exposed non-tumorous livers [As-Normal (AsN)], and arsenic-induced liver tumors (As-T ) in adult male mice. Lanes 1–3 show control liver; lanes 4–6 show arsenic exposed nontumorous normal liver; and lanes 7–9 show arsenic-induced HCC. The apparent kDa values were AFP ≈ 75 kDa, K-ras ≈28 kDa, c-Myc ≈70 kDa ER-α ≈ 70 kDa, cyclin D1 ≈35 kDa, cdk4 ≈33 kDa, PCNA ≈36 kDa, cytokeratin 8 ≈54 kDa. PAI-1 ≈47 kDa, EGFR ≈200 kDa, and β-actin ≈43 kDa.

We also performed Western blot analysis on selected gene products (Figure 3). Western blot analysis largely confirmed the gene expression results and clearly showed the increases in the expression of potential oncogenes AFP, K-ras, and c-Myc in both arsenic-exposed nontumorous livers and arsenic-induced HCC. The cell cycle regulators, such as ER-α, the ER-α linked cyclin D1, cyclin-dependent kinase 4 (cdk4), and proliferating cell nuclear antigen (PCNA), all followed this pattern of high in tumor tissues, intermediate in tumor-surrounding livers and low in control liver. The HCC biomarkers cytokeratin 8 and plasminogen activator inhibitor 1 (PAI-1) were all significantly increased in arsenic-induced HCC, and also higher in arsenic-exposed tumor-surrounding tissues. Endothelial growth factor receptor (EGFR) was down-regulated in arsenic-induced HCC. The expression of β-actin was consistent among lanes.

## Discussion

Our most recent transplacental arsenic carcinogenesis study (Waalkes et al. 2004b) confirmed the hepatocarcinogenic potential of arsenic in adult male mice after transplacental exposure (Waalkes et al. 2003), and it also provided an opportunity for a genomewide gene expression analysis. The 22K mouse chip array revealed several novel pathways and gene expression alterations associated with arsenic-induced HCC and in arsenic-exposed nontumorous normal liver samples, which were not detected in previous limited gene expression analysis. Liver is a major target organ for arsenic in rodents and in humans (Liu et al. 2000; 2002; Santra et al. 2000). In other studies, HCC formation has been associated with arsenic exposure in humans (Centeno et al. 2002; Chen et al. 1997; Chiu et al. 2004; Zhou et al. 2002). Therefore, the present gene profiling study in mice of a concordant human target tissue is noteworthy because it clearly demonstrates that multiple genetic events occurred during the carcinogenic process in the liver induced by *in utero* exposure to arsenic. It is reasonable to anticipate a complicated series of genetic interactions in arsenic-induced carcinogenesis, which this study certainly shows. However, the present results also provide a variety of candidate genes to investigate in greater detail, such as with time-course experiments, to define events early after transplacental arsenic exposure. Furthermore, recent evidence indicates that early life chemical exposures may aberrantly “reprogram” gene expression patterns, thus resulting in altered growth responses and cancer later in life, as with diethylstilbestrol and uterine cancer (Cook et al. 2005). Consistent with this concept, the altered gene expression patterns seen in adults after *in utero* arsenic exposure could have resulted from the early life reprogramming during a critical stage in development.

Clustering analysis clearly shows that the gene changes in HCC and normal liver samples taken from mice exposed to arsenic *in utero* are for the most part directionally consistent but with quantitatively greater changes in the arsenic-induced tumors. The gene expression alterations associated with arsenic hepatocarcinogenesis have an important impact on as many as 50 biological networks shown by Ingenuity Pathways Analysis to be significantly altered or interrupted. The observation that a large number of graded gene expression changes occurred both in nontumorous and in tumorous liver samples from adults exposed to arsenic *in utero* is important because it implies that complex changes initiated during gestation appear to have persisted into adulthood. Some of those alterations are very likely relevant to the carcinogenic process. The complex nature of this arsenic-induced response argues against mutational activation of a few key pathways and perhaps points toward other mechanisms for persistent gene expression change. For example, the central network role for c-Myc seen in the present study is consistent with *c-myc* activation in arsenic-transformed rat liver cells (Chen et al. 2001b), in hamster embryo cells (Takahashi et al. 2002), and in murine fibroblasts (Trouba et al. 2000). This central network role also agrees with that suggested by results from arsenic-induced skin tumors (Germolec et al. 1998) and from HCC induced by *in utero* arsenic exposure (Liu et al. 2004). Overexpression of *c-myc* may be caused by arsenic-induced DNA hypomethylation of the *c-myc* promoter region (Takahashi et al. 2002). The activation of *c-myc* oncogene can in turn result in many other gene expression changes. For example, the increased expression of the *N-myc* downstream regulated gene 1 (NDRG1) is associated with HCC formation. The increased expression of stearoyl-CoA desaturase-1 (SCD-1) plays a key role in lipogenic gene expression and in metabolic syndrome, which coincides with marked elevations of lipoprotein lipase (Lpl, 33-fold) and cytosolic acyl-CoA thioesterase-1 (Cte1, 5-fold) in arsenic-induced HCC. This increased expression could also be important for arsenic-induced hepatic steatosis (Chen et al. 2004). Interestingly, in this network, the increase in C-myc is associated with a decrease in metallothionein-1 expression (MT-1), which was confirmed by RT-PCR analysis. MT is an adaptive protein in response to arsenic exposure (Liu et al. 2000). The decrease in MT could be a marker for liver tumor progression because MT is poorly expressed in both mouse and human HCC (Waalkes et al. 1996). Determining the changes that are specific to arsenic and the changes that are part of the potentially autonomous process of liver oncogenesis will be a major challenge, but the present work opens various avenues for further study.

In addition to *c-myc* overexpression, the activation of various oncogenes such as *k-ras* and *c-met* was also evident following trans-placental arsenic exposure, as seen with arsenic-transformed rat liver cells (Chen et al. 2001a). Overexpression of *k-ras* is also associated with arsenic-induced malignant transformation in human prostate epithelial cells (Benbrahim-Tallaa et al. 2005). *Ha-ras* mutation and over-expression, as in genetically altered Tg.AC mice, is associated with arsenic-induced co-promotion of skin tumors (Germolec et al. 1998). In a fashion similar to *c-myc*, DNA hypomethylation could be responsible for *ras* oncogene overexpression, and chronic arsenic exposure *in vivo* appears to induce a loss of methylation at several cytosine sites within the promoter region of the hepatic *Ha-ras* gene in mice (Okoji et al. 2002). The Ras-mediated signal transduction molecule Rhou (ras homolog gene family U) was also increased 3- to 4-fold in arsenic-exposed tissues in the present study. The HCC biomarkers α-fetoprotein, plasminogen activator inhibitor 1 (PAI-1), cytokeratin 8, cytokeratin 18, and cytokeratin 19, as well as the neoplastic progression protein-3, were all significantly increased, consistent with HCC pathobiology and previous findings with arsenic (Liu et al. 2004). Conversely, the tumor suppressor genes *BRCA1* and *BRCA2* were decreased. BRCA1 protein interacts directly with ER-α, thereby resulting in inhibition of estradiol-stimulated ER-α transcriptional activity (Ma et al. 2005). Thus, down-regulation of BRCA1 and BRCA2 could play a role in the overexpression of ER-α and ER-α–linked gene expression observed in this model of arsenic carcinogenesis (Waalkes et al. 2004a).

The tumors (in the liver, ovary, adrenal, lung) and proliferative lesions (in the uterus and oviduct) induced by *in utero* arsenic exposure can all similarly be induced by estrogenic carcinogens (Waalkes et al. 2003; 2004b). Indeed, prior results demonstrated the over-expression of ER-α with a nuclear localization (active form) in adult male liver after *in utero* arsenic exposure (Waalkes et al. 2004a; Chen et al. 2004), a result confirmed by Western blot analysis in the present study. This finding leads to the hypothesis that arsenic could somehow produce estrogenic-like effects, possibly directly or indirectly through stimulation of ER-α, thus resulting in tumor formation (Waalkes et al. 2004a). Overexpression of ER-α could be caused by arsenic-induced DNA hypomethylation of the promoter region of the ER-α gene (Chen et al. 2004; Waalkes et al., 2004a). Such overexpression is associated with the feminization pattern of liver metabolic enzymes in male liver bearing arsenic-induced HCC (Liu et al. 2004; Waalkes et al. 2004a). In the present study, marked elevation of the female predominant CYP2A4, CYP2B9, and CYP2D9 and significant decreases in male dominant CYP7B1, CYP2F2, and CYP41 were evident following transplacental arsenic exposure in male offspring, consistent with this hypothesis. The contribution of ER-α signaling pathways to arsenic-induced HCC could be multifactorial, including the increased trefoil factor-3 (TFF3), cyclin D1, and the CCN family members cysteine-rich 61 (Cyr61) and connective tissue growth factor (CTGF). Overexpression of TFF3 is proposed to be a critical process in hepatocarcinogenesis (Okada et al. 2005), and overexpression of Cyr61 and CTGF is associated with recurrence and metastasis of HCC (Zeng et al. 2004).

Estrogen-induced progression through the G_1_ phase of the cell cycle is preceded by increased expression of the G_1_-phase regulatory proteins c-Myc and Cyclin D1 (Prall et al. 1998). Cyclin D1 overexpression is important for HCC formation and is considered a hepatic oncogene (Deane et al. 2001). Overexpression of cyclin D1 has been reported in arsenic-transformed cells (Chen et al. 2001b), during arsenic-induced skin co-carcinogenicity in mice (Rossman et al. 2001), in dimethylarsinic acid–induced bladder proliferative lesions in rats (Wei et al. 2002), in chronic arsenate-exposed rats (Cui et al. 2004), and in the livers of mice bearing HCC induced by *in utero* exposure to arsenic (Liu et al. 2004). Cyclin D1 overexpression, together with other positive cell cycle regulators such as cyclin E, cdk4, cdkn2a, cdkn2b, and PCNA was evident in arsenic-exposed tissues in the present study. Thus, cell cycle dysregulation, as manifested in cyclin D1 overexpression, appears to be a consistent event in arsenic-induced oncogenesis. In comparison, expression of the negative cell cycle regulator p16 was decreased, consistent with prior observations with arsenic (Chen et al. 2001a; Liu et al. 2004). In addition to these cell cycle regulators, annexin A2 was also dramatically increased (48-fold) in arsenic-induced HCC. Annexins belong to a family of the calcium-dependent phospholipid binding proteins and are substrates of receptor tyrosine kinases. Annexin A2 overexpression is common in various carcinomas (Emoto et al. 2001), and annexin A2 can interact with c-Myc in increasing cell proliferation (Mickleburgh et al. 2005). Dysregulation of the IGF axis, including IGF-1 and IGF-2, and IGF binding proteins, was evident in our prior work (Liu et al. 2004) and in our present studies. A decrease in IGF-1 and an increase in IGFBP1 in arsenic-exposed livers are similar to findings with the nongenotoxic model carcinogens such as oxazepam and Wyeth-14,643 (Iida et al. 2003). Dysregulation of the IGF axis may also well contribute to uncontrolled cell proliferation and hepatocarcinogenesis (Scharf and Braulke 2003).

The expression of oxidative stress-related genes is associated with arsenic toxicity and cell proliferation (Liu et al. 2001; Pi et al. 2003). For example, overexpression of early growth protein 1 (EGR1) is associated with arsenic-induced proliferation in urinary bladder cells (Simeonova et al. 2000) and in arsenic-induced HCC (Liu et al. 2004). The activation of transcription factor Nrf2 by arsenic and the Nrf2-mediated overexpression of antioxidants such as Cu,Zn superoxide dismutase (SOD1) is evident in arsenic-exposed cells (Pi et al. 2003). Expression of SOD1 and the Cu-binding protein ceruloplasmin was increased in arsenic-exposed liver and in arsenic-induced liver tumors in the present work. The increases in these acute phase proteins may have clinical relevance because increased ceruloplasmin (Bencko et al. 1988) and SOD1 (Lu et al. 2001) have been reported in arsenic-exposed humans. Heme oxygenase-1, a biomarker for acute arsenic-induced oxidative stress (Liu et al. 2001a; NRC 1999), was not significantly altered in the present study. In comparison, GST-alpha, GST-mu, GST-pi, and GST-theta expressions were all increased following *in utero* arsenic exposure, similar to previous observations in chronic arsenic exposure settings (Liu et al. 2000; Lu et al. 2001; Xie et al. 2004). Increased GSTs likely play a role in conjugation and detoxication of lipid peroxides produced by arsenic, but they also play a role for cellular efflux of arsenic via MRP pumps (Leslie et al. 2004; Liu et al. 2001b). The increased demand for GSH utilization may activate the use of homocysteine for GSH synthesis, rather than for *S*-adenosylmethionine (SAM), and, consequently, disrupt cellular methyl homeostasis (Lu et al. 2002). In this regard, expression of betaine-homocysteine methyltransferase was markedly decreased, and it is a key enzyme in SAM recycling. This disruption of SAM production may contribute to a cellular environment conducive to errors in DNA methylation, which can be a contributing factor to aberrant gene expression.

Both β-catenin and E-cadherins are important cell–cell communication molecules. The overexpression of β-catenin is linked to cyclin D1 overexpression in HCC (Iida et al., 2003). Furthermore, β-catenin can also affect tumor progression by stimulating proliferation, and overexpression is associated with a poor prognosis for patients with HCC. E-cadherin is a cell–cell adhesion molecule that plays a key role in the development and maintenance of cell polarity. Dysregulation of E-cadherin is also associated with HCC (Calvisi et al. 2004). Nidogen-1 acts as a bridge between the extra-cellular matrix molecules laminin-1 and collagen IV, and it participates in the assembly of the basement membranes. In the present study, nidogen 1 (50-fold), β-catenin (7-fold), and E-cadherin (11-fold) were all dramatically increased in transplacental arsenic-induced HCC and in arsenic-exposed liver tissues. Conversely, the prolactin receptor and EGFR were decreased in arsenic-induced HCC, thereby suggesting that the dysregulation of cell–cell communications and signal transduction pathways could be another important aspect of arsenic hepatocarcinogenesis, as has been proposed for liver cancer in general.

In summary, we used a genomewide analysis to dissect further the toxicogenomics changes of transplacental arsenic hepatocarcinogenesis in the mouse. These findings clearly demonstrate that arsenic carcinogenesis in the liver involves a complex interplay between multiple genetic events, including stimulation of oncogene expression, liver feminization, cell cycle dysregulation, and disruption of cell–cell communication. This study indicates that early life exposure to arsenic during a critical stage in development may have resulted in aberrant genetic reprogramming that leads to events associated with hepatocarcinogenesis later in life. Finally, the present results highlight the importance of protecting pregnant women from excessive arsenic exposure.

## Figures and Tables

**Table 1:** Real-time RT-PCR and microarray analysis of liver samples from adult male C3H mice exposed to arsenic *in utero*.

			As-Normal		As-HCC	
Gene categories^a^	Accession no.^a^	Control (PCR)	PCR	Array-fold	PCR	Array-fold
Oncogenes and HCC-related genes						
*alpha-fetoprotein*	V00743	1.0 ± 0.2	2.3 ± 0.5	NA	19.1 ± 5.4*	NA
*c-myc*	X01023	1.0 ± 0.2	2.2 ± 0.5	1.67*	3.1 ± 0.5*	2.36*
*c-met*	Y00671	1.0 ± 0.2	2.4 ± 0.6	1.03	3.4 ± 0.8*	1.26*
*K-ras*	U49448	1.0 ± 0.2	1.4 ± 0.4	1.41	2.5 ± 0.6*	1.54*
*PAI-1,* plasminogen activator inhibitor-1	M33960	1.0 ± 0.3	2.7 ± 0.9	NA	9.2 ± 2.9*	NA
*Cytokeratin-8*	X12789	1.0 ± 0.2	1.6 ± 0.2	NA	6.0 ± 1.3*	NA
*Cytokeratin-18*	M11686	1.0 ± 0.3	1.3 ± 0.3	1.04	2.9 ± 0.4*	2.34*
*Cytokeratin-19*	NM_008471	1.0 ± 0.4	12.1 ± 4.7*	1.53*	11.1± 5.4*	1.24
*BRCA1*	U31625	1.0 ± 0.3	0.7 ± 0.1	1.08	0.4 ± 0.1*	0.81*
*BRCA2*	U65594	1.0 ± 0.1	0.8 ± 0.1	NA	0.7 ± 0.1*	NA
*Npn3,* neoplastic progression protein-3	Z31362	1.0 ± 0.4	1.0 ± 0.3	1.05	3.1 ± 0.8*	2.41*
Cell cycle regulators and IGFs						
*Cyclin D1*	M64403	1.0 ± 0.2	4.8 ± 1.0*	1.62*	5.1 ± 0.9*	1.51*
*Cyclin E*	X75888	1.0 ± 0.1	4.4 ± 1.5*	1.19*	5.2 ± 1.3*	1.31*
*Cdk2na*	NM_009877	1.0 ± 0.2	7.1 ± 1.9*	1.01	13.9 ± 3.8*	1.20
*Cdk2nb*	AF_059567	1.0 ± 0.3	2.3 ± 0.6	NA	6.3 ± 1.8*	NA
*Cdk4*	L01640	1.0 ± 0.3	2.5 ± 0.5	1.53*	4.4 ± 0.2*	1.76*
*PCNA*	X53068	1.0 ± 0.1	2.5 ± 0.8	1.73*	2.6 ± 0.5*	1.76*
*P16*	U79632	1.0 ± 0.3	0.6 ± 0.2	0.85	0.6 ± 0.1*	0.83*
*IGF-1*	X04480	1.0 ± 0.1	0.7 ± 0.1	0.65*	0.6 ± 0.1*	0.53*
*IGF-2*	M14951	1.0 ± 0.2	3.3 ± 2.1	3.77*	4.4 ± 2.0*	4.21*
*IGFBP1*	X81579	1.0 ± 0.3	2.7 ± 0.5*	1.15	9.1 ± 3.2*	4.18*
*IGFBP3*	X81581	1.0 ± 0.4	2.9 ± 0.8*	0.88	2.9 ± 0.8*	0.88
*IGFBP5*	X81583	1.0 ± 0.2	4.6 ± 1.5*	0.94	2.4 ± 0.9*	0.91
Stress-related genes						
*GST-alpha4*	AK008490	1.0 ± 0.4	2.5 ± 0.6*	1.94*	2.5 ± 0.6*	2.16*
*GST-mu*	J03953	1.0 ± 0.4	3.5 ± 0.8*	1.54*	4.8 ± 1.1*	1.26*
*GST-theta*	X98055	1.0 ± 0.5	3.5 ± 0.8*	1.14	3.1 ± 0.9*	1.31*
*GST-pi*	NM_013541	1.0 ± 0.4	2.5 ± 0.6*	2.23	2.5 ± 0.6*	1.11
*EGR1,* early response protein-1	M20157	1.0 ± 0.5	3.5 ± 0.8*	1.24*	3.1 ± 0.9*	5.20*
*SOD-1*	M01143	1.0 ± 0.4	2.5 ± 0.6*	NA	2.5 ± 0.6*	NA
*Ceruloplasmin*	U49430	1.0 ± 0.1	4.1 ± 1.0*	1.02	4.3 ± 1.5*	1.14*
*HO-1,* heme oxygenase-1	M33203	1.0 ± 0.1	1.0 ± 0.2	1.51*	0.9 ± 0.2	1.23*
*MT-1*	BC027262	1.0 ± 0.2	0.6 ± 0.3	0.50*	0.5 ± 0.1*	0.59*
Genes for metabolic enzymes						
*CYP2A4*	J03549	1.0 ± 0.1	3.1 ± 0.9*	1.05	25.3 ± 8.7*	2.51*
*CYP2F2*	M77497	1.0 ± 0.2	1.0 ± 0.2	1.12	0.5 ± 0.2*	0.68*
*CYP2B9*	M21855	1.0 ± 0.4	1.9 ± 0.6	0.85	2.6 ± 1.1*	0.43
*CYP2D9*	M27168	1.0 ± 0.4	5.3 ± 1.0*	1.15*	4.7 ± 1.2*	1.06
*CYP3A41*	NM_017396	1.0 ± 0.5	0.4 ± 0.2	0.55*	0.4 ± 0.1*	0.57*
*CYP7B1*	U36993	1.0 ± 0.3	0.7 ± 0.3	0.82*	0.5 ± 0.1*	0.39*
*Akr1c18,* aldoketo reductase 1c18	NM_134066	1.0 ± 0.5	2.5 ± 0.8	1.39	61.7 ± 27.8*	15.3*
*HSD17* β*7*	NM_010476	1.0 ± 1.4	3.1 ± 0.4*	0.92	2.8 ± 0.4	0.99
*TFF3,* trefoil factor 3	NM_011575	1.0 ± 0.1	3.2 ± 1.0*	5.82*	6.3 ± 1.8*	10.9*
*Cyr61,* cysteine-rich protein 61	NM_010516	1.0 ± 1.0	1.7 ± 0.3	0.85	4.9 ± 1.7*	1.17
*Lp1,* lipoprotein lipase	NM_008509	1.0 ± 0.1	12.1 ± 5.1*	4.61*	33.2 ± 13.1*	9.21*
*Cte1,* cytosolic acyl-CoA thioestase1	NM_012006	1.0 ± 0.2	2.7 ± 0.8	1.29*	4.8 ± 1.8*	2.18*
*Pmsc3,* proteasome 26S subunit, ATPase3	D49686	1.0 ± 1.1	2.6 ± 0.4	1.62*	4.1 ± 1.1*	1.61*
*BHMT,* homocysteine methyltransferase	AF033381	1.0 ± 0.1	0.7 ± 0.1	0.84*	0.6 ± 0.1*	0.63*
*Saa3,* serum amyloid 3	NM_011315	1.0 ± 0.3	2.2 ± 0.7	0.23*	0.3 ± 0.1*	0.05*
*SULT-X2*	AF026075	1.0 ± 0.1	0.1 ± 0.0*	0.21*	0.0 ± 0.0*	0.16*
Cell communication and signal transduction						
*Annexin A2*	M14044	1.0 ± 0.3	9.0 ± 2.9*	2.30*	48.5 ± 16.9*	8.64*
*Nid1,* nidogen 1	NM_010917	1.0 ± 0.2	3.1 ± 1.1	2.60*	56.2 ± 9.8*	10.5*
β*-catenin*	NM_007614	1.0 ± 0.3	4.2 ± 0.8*	2.01*	6.9 ± 2.4*	2.03*
*E-cadherin*	NM_009864	1.0 ± 0.3	4.4 ± 1.5*	1.24*	11.2 ± 4.5*	4.34*
*Ptgs-2,* prostaglandin-endoperoxide synthase 2	NM_011198	1.0 ± 0.4	2.6 ± 1.0	1.12	5.6 ± 2.5*	1.18
*Rhou,* ras homolog gene family U	NM_133955	1.0 ± 0.2	3.1 ± 0.8*	NA	3.9 ± 1.0*	NA
*CTGF,* fibronectin	NM_022266	1.0 ± 0.3	2.7 ± 0.6	2.50*	3.7 ± 0.8*	1.90*
*Prlr,* prolactin receptor	NM_011169	1.0 ± 0.4	0.4 ± 0.2	0.37*	0.4 ± 0.1*	0.23*
*Egfr,* epidermal growth factor receptor	NM_007912	1.0 ± 0.2	1.1 ± 0.2	0.71*	0.5 ± 0.1*	0.63*

NA, same gene access clone is not available. Data are mean ± SEM of 5–7 individual animals.

a Gene names, symbols, and accession numbers are from GenBank (http://www.ncbi.nih.gov/GenBank/).

* Significantly different from controls *p* < 0.05.
